# Retrospective analysis of ocular adverse events with Clobetasol

**DOI:** 10.1111/dth.15574

**Published:** 2022-05-29

**Authors:** Yu Wang, Joseph L. Jorizzo

**Affiliations:** ^1^ Department of Dermatology Wake Forest University Winston‐Salem North Carolina USA; ^2^ Department of Dermatology Weill Cornell Medicine New York USA

Psoriasis is a chronic autoinflammatory skin disease that affects 2% of the population worldwide.^1^, ^2^ Psoriasis has various clinical manifestations, the most common one being plaque psoriasis.^1^, ^2^ When it is untreated, the complications include chronic pain, bleeding, pruritus, and depression.^3^ The treatment of psoriasis is often determined by clinical severity of the lesions and the extent/percentage of the affected body surface.^3^ For mild‐to‐moderate psoriasis, high potency topical corticosteroids (TCS), vitamin D analogues, and phototherapy are often the first‐line treatment.^3^ However, one significant concern about high potency TCS use is systemic absorption, which lead to an increased rate of cataracts and glaucoma, which has been associated with oral cortisol steroid use.^4^ Since clobetasol is one of the most used high‐potency TCS for the treatment of psoriasis, our goal was to analyze the rate of cataracts and glaucoma reported to the FDA in association with topical clobetasol usage versus systemic therapies such as methotrexate and adalimumab when used in psoriasis treatment.

Ocular adverse events associated with clobetasol, methotrexate, and adalimumab that were reported to the Federal Drug Administration (FDA) Adverse Event Reporting System (FAERS) Database were gathered and assessed.^5^

In total, 3466 total adverse events associated with clobetasol were reported to FAERS. Of these events, 19 (0.55%) were cataracts and 22 (0.63%) were glaucoma. A total of 7359 adverse events reported were associated with methotrexate for patients being treated for dermatologic conditions only, of which 140 (1.91%) were cataracts and 382 (5.19%) were glaucoma. A total of 41,737 adverse events reported were associated with adalimumab for patients being treated for dermatologic conditions only, of which 198 (0.47%) were cataracts and 74 (0.18%) were glaucoma.

According to the FAERS data, patients treated with clobetasol have an overall low incidence of cataracts (0.55%) and glaucoma (0.63%) as adverse events. Worldwide, the incidence of cataracts is approximately 2.5% in those aged of 40–49 years, 6.8% in those aged of 50–59 years, 20.0% in those aged of 60–69 years, 42.8% in those aged of 70 to 79 years, and 68.3% in those aged greater than 80 years.^6^ Globally an estimated 3.5% of people aged 40–80 are diagnosed with glaucoma.^7^ When compared to the incidence of cataracts and glaucoma, the incidence of cataracts and glaucoma is much lower with less than 0.7% for both among all reported adverse events associated with clobetasol. Furthermore, patients being treated for psoriasis with clobetasol did not demonstrate a significant statistical difference in the incidence of cataracts when compared to those being treated with methotrexate (0.55% vs. 1.91%, *P* > 0.05) or adalimumab (0.55% vs. 0.47%, *P* > 0.05). Patients being treated for psoriasis with clobetasol had a lower rate in the incidence of glaucoma when compared to those being treated with methotrexate (0.63% vs. 5.19%, *P* < 0.05) and no statistically significant difference when compared to adalimumab (0.63% vs. 0.18%, *P* > 0.05). A recent review of all case reports regarding TCS associated ocular adverse events by *Daniel* et al.^8^ also supports the FDA data. A comprehensive literature search was conducted on PUBMED, Google, and Cochrane databases using the search criteria: topical corticosteroids and ocular side‐effects, glaucoma, and cataracts. No cases reports of glaucoma or cataracts associated with high potency TCS applied to non‐periorbital area were found (Table 1).^8^, ^9^, ^10^, ^11^, ^12^, ^13^, ^14^, ^15^, ^16^, ^17^, ^18^, ^19^, ^20^, ^21^, ^22^ Thus, the findings support the understanding that the application of appropriately prescribed TCS to areas other than periorbital skin is unlikely to result in ocular disease.^8^

**TABLE 1:** Previous studies on topical steroids which reported cataract and glaucoma with mentioning of duration and location of use.^9^, ^10^, ^11^, ^12^, ^13^, ^14^, ^15^, ^16^, ^17^, ^18^, ^19^, ^20^, ^21^, ^22^

Author	Age	Sex	Duration of treatment with topical steroids	Site of application	Indication	Glaucoma	Cataracts
Cubey	22	Male	Daily for 7 years	Face and eyelids	Facial eczema	Yes	No
Ross et al	42	Male	twice weekly for 2 years	Face, neck, chest and arms	Atopic eczema	Yes	No
Aggarwal et al	24	Male	2 years	Face and eyelids	Eczema	Yes	No
Aggarwal et al	23	Male	Intermittently for 12 years	Periorbital skin	Atopic eczema	Yes	Yes
Aggarwal et al	25	Male	4 years	Periorbital skin	Severe atopic eczema	Yes	Yes
Vie	29	Female	“Many years”	Eyelids	Daily for many years	Yes	No
Nielsen	68	Female	Several times a day	Bilateral eyelids	Periorbital eczema	Yes	No
Nielsen	80	Female	3 times per day for 3 years	Bilateral periorbital	Periorbital dermatosis	Yes	No
Eisenlohr	33	Female	Daily for 3–5 years	Eyelids	Irritation from cosmetics	Yes	No
Sahni et al	29	Female	Since childhood (16–25 years total)	Face, periorbital, flexures, body and limbs	Atopic dermatitis	Yes	No
Garrot et al	40	Male	“chronic”	Eyelids	Psoriasis, steroid rosacea	Yes	No
Michaeli‐Cohen	33	Female	Over 15 years	Hands and face	Atopic dermatitis	Yes	Yes
Michaeli‐Cohen	45	Male	20 years	Hands and face	Atopic dermatitis	Yes	Yes
Ross et al	42	Male	Prescribed 5 times over 2 year period; applied twice weekly	Face, neck, chest and arms	Atopic eczema	Yes	No
Howell	Unknown	Unknown	Unknown	Eyelids	Blepharitis	Yes	No
Kabata et al	37	Male	24 years	Face, neck, chest and arms	Atopic dermatitis	Yes	No
Katsushima	Unknown	Unknown	2 years	Periorbital	Vitiligo	No	Yes
Katsushima	Unknown	Unknown	Unknown	Periorbital	Atopic dermatitis	No	Yes
Katsushima	Unknown	Unknown	Unknown	Periorbital	Atopic dermatitis	No	Yes
McLean et al	30	Male	Unknown	Face, chest and arms	Discoid eczema	Yes	No
Sim et al	35	Male	3 times daily for 20 years	Face	Eczema	Yes	No

Of note, it is essential to educate patients about avoiding application of clobetasol to the face, as it can cause perioral dermatitis/steroid rosacea.^8^ However, the data in the current literature and our analysis of FAERS suggests that treatment with clobetasol, when used properly on scalp and thicker body lesions, does not increase the rate of cataracts and glaucoma. In conclusion, systemic absorption is unlikely to lead to ocular adverse events and should be kept in consideration when providing patient care.

## CONFLICT OF INTEREST

Dr. Wang and Dr. Jorizzo have no conflicts of interest relevant to the content of the submission. This work has not been previously published.

## Data Availability

The data that support the findings of this study are available in FDA FAERS Database at https://www.fda.gov/drugs/questions‐and‐answers‐fdas‐adverse‐event‐reporting‐system‐faers/fda‐adverse‐event‐reporting‐system‐faers‐public‐dashboard, reference number [5]. These data were derived from the following resources available in the public domain: https://www.fda.gov/drugs/questions‐and‐answers‐fdas‐adverse‐event‐reporting‐system‐faers/fda‐adverse‐event‐reporting‐system‐faers‐public‐dashboard
